# Supplementary material for: HIVprotI: an integrated web based platform for prediction and design of HIV proteins inhibitors
Source: J Cheminform. 2018 Mar 9;10:12. doi: 10.1186/s13321-018-0266-y (PMC5845081; doi:10.1186/s13321-018-0266-y)
Supplement: Supplementary file 2 — Additional file 2. Source code of HIVProtI web server. [file 13321_2018_266_MOESM2_ESM.zip › HIVprotI_Source-code/hivprotI/weka-3-6-6/doc/weka/classifiers/trees/adtree/TwoWayNominalSplit.html]

TwoWayNominalSplit


---


|  |  |  |
| --- | --- | --- |
| **Overview** \| **Package** \| **Class** \| **Tree** \| **Deprecated** \| **Index** \| **Help** \| **Weka's home** | |  |
| **PREV CLASS**   **NEXT CLASS** | **FRAMES**    **NO FRAMES**     **All Classes** |  |
| SUMMARY: NESTED \| FIELD \| CONSTR \| METHOD | DETAIL: FIELD \| CONSTR \| METHOD |  |


---


## weka.classifiers.trees.adtree Class TwoWayNominalSplit

```
java.lang.Object
  weka.classifiers.trees.adtree.Splitter
      weka.classifiers.trees.adtree.TwoWayNominalSplit
```

**All Implemented Interfaces:**: java.io.Serializable, java.lang.Cloneable, RevisionHandler

---

``` public class TwoWayNominalSplit extends Splitter ```

Class representing a two-way split on a nominal attribute, of the form:
either 'is some\_value' or 'is not some\_value'.

**Version:**
:   $Revision: 1.6 $

**Author:**
:   Richard Kirkby (rkirkby@cs.waikato.ac.nz)

**See Also:**: Serialized Form

---

**Field Summary**

**Fields inherited from class weka.classifiers.trees.adtree.Splitter**

`orderAdded`


**Constructor Summary**

`TwoWayNominalSplit(int _attIndex,
int _trueSplitValue)`
  
          Creates a new two-way nominal splitter.


| **Method Summary** | |
| --- | --- |
| `java.lang.String` | `attributeString(Instances dataset)`             Gets the string describing the attributes the split depends on. |
| `int` | `branchInstanceGoesDown(Instance inst)`             Gets the index of the branch that an instance applies to. |
| `java.lang.Object` | `clone()`             Clones this node. |
| `java.lang.String` | `comparisonString(int branchNum, Instances dataset)`             Gets the string describing the comparision the split depends on for a particular branch. |
| `boolean` | `equalTo(Splitter compare)`             Tests whether two splitters are equivalent. |
| `PredictionNode` | `getChildForBranch(int branchNum)`             Gets the child for a branch of the split. |
| `int` | `getNumOfBranches()`             Gets the number of branches of the split. |
| `java.lang.String` | `getRevision()`             Returns the revision string. |
| `ReferenceInstances` | `instancesDownBranch(int branch, Instances instances)`             Gets the subset of instances that apply to a particluar branch of the split. |
| `void` | `setChildForBranch(int branchNum, PredictionNode childPredictor)`             Sets the child for a branch of the split. |

**Methods inherited from class java.lang.Object**

`equals, getClass, hashCode, notify, notifyAll, toString, wait, wait, wait`

**Constructor Detail**

### TwoWayNominalSplit

```
public TwoWayNominalSplit(int _attIndex,
                          int _trueSplitValue)
```

:   Creates a new two-way nominal splitter.

    **Parameters:**: `_attIndex` - the index of the attribute this split depeneds on: `_trueSplitValue` - the attribute value that the splitter splits on


**Method Detail**

### getNumOfBranches

```
public int getNumOfBranches()
```

:   Gets the number of branches of the split.

    :   **Specified by:**: `getNumOfBranches` in class `Splitter`
    :   **Returns:**: the number of branches (always = 2)

---


### branchInstanceGoesDown

```
public int branchInstanceGoesDown(Instance inst)
```

:   Gets the index of the branch that an instance applies to. Returns -1 if no branches
    apply.

    :   **Specified by:**: `branchInstanceGoesDown` in class `Splitter`
    :   **Parameters:**: `inst` - the instance **Returns:**: the branch index

---


### instancesDownBranch

```
public ReferenceInstances instancesDownBranch(int branch,
                                              Instances instances)
```

:   Gets the subset of instances that apply to a particluar branch of the split. If the
    branch index is -1, the subset will consist of those instances that don't apply to
    any branch.

    :   **Specified by:**: `instancesDownBranch` in class `Splitter`
    :   **Parameters:**: `branch` - the index of the branch: `instances` - the instances from which to find the subset **Returns:**: the set of instances that apply

---


### attributeString

```
public java.lang.String attributeString(Instances dataset)
```

:   Gets the string describing the attributes the split depends on.
    i.e. the left hand side of the description of the split.

    :   **Specified by:**: `attributeString` in class `Splitter`
    :   **Parameters:**: `dataset` - the dataset that the split is based on **Returns:**: a string describing the attributes

---


### comparisonString

```
public java.lang.String comparisonString(int branchNum,
                                         Instances dataset)
```

:   Gets the string describing the comparision the split depends on for a particular
    branch. i.e. the right hand side of the description of the split.

    :   **Specified by:**: `comparisonString` in class `Splitter`
    :   **Parameters:**: `branchNum` - the branch of the split: `dataset` - the dataset that the split is based on **Returns:**: a string describing the comparison

---


### equalTo

```
public boolean equalTo(Splitter compare)
```

:   Tests whether two splitters are equivalent.

    :   **Specified by:**: `equalTo` in class `Splitter`
    :   **Parameters:**: `compare` - the splitter to compare with **Returns:**: whether or not they match

---


### setChildForBranch

```
public void setChildForBranch(int branchNum,
                              PredictionNode childPredictor)
```

:   Sets the child for a branch of the split.

    :   **Specified by:**: `setChildForBranch` in class `Splitter`
    :   **Parameters:**: `branchNum` - the branch to set the child for: `childPredictor` - the new child

---


### getChildForBranch

```
public PredictionNode getChildForBranch(int branchNum)
```

:   Gets the child for a branch of the split.

    :   **Specified by:**: `getChildForBranch` in class `Splitter`
    :   **Parameters:**: `branchNum` - the branch to get the child for **Returns:**: the child

---


### clone

```
public java.lang.Object clone()
```

:   Clones this node. Performs a deep copy, recursing through the tree.

    :   **Specified by:**: `clone` in class `Splitter`
    :   **Returns:**: a clone

---


### getRevision

```
public java.lang.String getRevision()
```

:   Returns the revision string.

    :   **Returns:**: the revision


---


|  |  |  |
| --- | --- | --- |
| **Overview** \| **Package** \| **Class** \| **Tree** \| **Deprecated** \| **Index** \| **Help** \| **Weka's home** | |  |
| **PREV CLASS**   **NEXT CLASS** | **FRAMES**    **NO FRAMES**     **All Classes** |  |
| SUMMARY: NESTED \| FIELD \| CONSTR \| METHOD | DETAIL: FIELD \| CONSTR \| METHOD |  |


---
